# The Relationship Between College Students’ Emotional Intelligence, Foreign Language Enjoyment, and L2 Willingness to Communicate: A Variable-Centered and Person-Centered Perspective

**DOI:** 10.3390/bs15111508

**Published:** 2025-11-06

**Authors:** Zheying Xiao, Jia Jia

**Affiliations:** 1 Faculty of Foreign Languages, Wenhua College, Wuhan 430074, China; xiaozheying@whc.edu.cn; 2 School of Foreign Languages, South China University of Technology, Guangzhou 510641, China

**Keywords:** emotional intelligence, foreign language enjoyment, L2 willingness to communicate, Chinese EFL learners in private colleges, person-centered analysis, latent profile analysis

## Abstract

In the field of second language acquisition, there is a growing recognition of the importance of emotional factors, particularly emotional intelligence (EI), in influencing learners’ willingness to communicate (WTC) in a second language (L2). However, previous studies have predominantly adopted a variable-centered approach, often overlooking individual heterogeneity and the mediating role of foreign language enjoyment (FLE) in the relationship between EI and WTC. To address these gaps, this study integrated variable-centered and person-centered approaches to examine 1111 students from Chinese private colleges, representing a distinct educational ecology. Questionnaires were used to measure EI, FLE, and L2 WTC, followed by mediation analysis and latent profile analysis (LPA). Results indicated that EI positively predicted WTC (*β* = 0.217, *p* < 0.001), with FLE partially mediating this relationship (indirect *β* = 0.135, *p* < 0.001), accounting for 38.3% of the total effect (*β* = 0.352, *p* < 0.001). LPA identified three learner profiles—“High EI–High Enjoyment,” “Moderate EI–Moderate Enjoyment,” and “Low EI–Low Enjoyment.” These profiles differed significantly in L2 WTC (*p* < 0.005), although the effect size was small (*η*^2^ = 0.002). The findings revealed that learners with higher emotional intelligence and enjoyment tended to report greater willingness to communicate. However, the overall effect was small, suggesting that emotional factors may serve as facilitators rather than decisive determinants of L2 communication. Despite the modest magnitude of these differences, the pattern highlights subtle yet meaningful emotional dynamics underlying L2 communication behavior. By integrating person- and variable-centered perspectives, this study contributes methodological refinement and provides cautiously framed pedagogical implications for fostering emotional engagement and communicative willingness among diverse L2 learners.

## 1. Introduction

Willingness to communicate (WTC) in a second language (L2) refers to an individual’s readiness to engage in L2 communication with a specific interlocutor at a particular time and in a particular context (MacIntyre et al., 1998). WTC is not only closely linked to developing of communicative competence, but is also widely regarded as a key indicator of successful second language acquisition (SLA) outcomes (L. Wei et al., 2022). In pursuit of understanding how L2 WTC can be fostered among college students, previous research has explored multiple predictors across individual, interactional, and contextual levels, including teacher support (Yin et al., 2024), language thinking patterns (B. B. Li et al., 2023), classroom social climate (Khajavy et al., 2016; H. Wang et al., 2021), and affective variables such as foreign language anxiety and enjoyment (Dewaele et al., 2018). However, findings across studies are not always convergent, and there is evidence that the relative contribution of these factors may vary by contexts and learner groups, suggesting that affective and ecological conditions surrounding learners need to be examined more closely rather than being treated as uniform.

With the growing influence of positive psychology in SLA (G. Li, 2024; Y. Q. Wang, 2023), emotional factors have received increasing attention as potential facilitators of L2 communication. Emotional intelligence (EI), broadly understood as one’s capacity to perceive, understand, and regulate emotions, has been linked to higher self-confidence, greater emotional regulation, and more adaptive classroom engagement, which in turn have been associated with greater willingness to communicate in the L2 (Thao et al., 2023). At the same time, foreign language enjoyment (FLE) has been identified as a proximal affective correlate of L2 WTC, in that learners who experience enjoyment in the language classroom tend to report a stronger inclination to initiate and sustain communication in the target language (Dewaele & Pavelescu, 2019). While these strands of work point to the importance of EI and FLE, the specific pathway through which EI may relate to L2 WTC via FLE has not yet been sufficiently clarified, particularly in terms of whether enjoyment functions as an intervening mechanism between learners’ emotional resources and their communicative readiness.

From a theoretical perspective, the present study draws on emotional intelligence theory (Salovey & Mayer, 1990), the control–value theory of achievement emotions (Pekrun, 2006) and an ecological view of L2 classrooms (Benner et al., 2008). Emotional intelligence theory conceptualizes EI as the ability to perceive, understand, and regulate emotions in oneself and others, providing a foundation for how learners manage affective experiences during L2 communication. Control–value theory posits that learners’ appraisals of control and value in achievement settings shape their achievement-related emotions, such as enjoyment, which in turn are linked to engagement tendencies, including communicative engagement in the classroom. Within this view, learners with higher EI, particularly in terms of monitoring and managing their own emotional states, may be more likely to experience positive achievement emotions such as enjoyment, which are themselves associated with higher reported WTC. In parallel, an ecological perspective on L2 communication (e.g., H. Wang et al., 2021; Khajavy et al., 2016) emphasizes that WTC emerges from the dynamic interplay between individual affective resources and the affordances and constraints of the immediate learning environment. This implies that the EI → FLE → WTC pathway may be shaped by contextual features of particular educational ecologies.

Despite these theoretical links, two important gaps remain. First, much of the existing evidence on EI, enjoyment, and WTC has been generated in general university settings (Wen & Clément, 2003; Zheng & Cheng, 2018), whereas comparatively little is known about how these relationships unfold in Chinese private colleges. Students in such institutions often learn in resource-constrained yet competitive environments, which may influence both their affective experiences in the L2 classroom and their willingness to take communicative risks (H. Wang, 2020). Whether the same emotional mechanisms apply in this ecology is therefore an open question. Second, most prior work has adopted a variable-centered approach, which is well suited to estimating overall relations between constructs but less informative about heterogeneity among learners (Howard & Hoffman, 2018). In particular, it remains unclear whether distinct constellations of emotional intelligence and enjoyment can be identified at the learner level, and whether such constellations are differentially associated with L2 WTC.

To address these issues, the present study focused on Chinese private college students as a specific group of English as a foreign language (EFL) learners and integrated both variable-centered and person-centered approaches. At the variable-centered level, we examined whether emotional intelligence predicts L2 WTC and whether foreign language enjoyment partially mediates this association, consistent with a control–value account. At the person-centered level, we used latent profile analysis to identify distinct EI–FLE learner profiles and compared their levels of L2 WTC, thereby situating communicative willingness within an ecological understanding of learner emotional profiles. By linking individual emotional resources, enjoyment in the classroom, and self-reported willingness to communicate, the study aims to clarify the emotional mechanisms that support L2 communication in this particular educational context and to provide cautiously framed pedagogical implications for fostering emotionally supportive classroom environments.

## 2. Emotional Intelligence and L2 Willingness to Communicate

Emotional intelligence was first introduced by Salovey and Mayer, who defined it as the ability to perceive, understand, and manage one’s own emotions and those of others to facilitate personal adaptation and development in social interactions (Mayer et al., 1993). Later conceptualizations, such as Bar-On’s model, further expanded EI to include a broad set of emotional, interpersonal, and personality-related competencies for coping with environmental demands (Dawda & Hart, 2000). While these early models emphasized ability-based perspectives, the present study adopted a trait emotional intelligence framework, operationalized through the TEIQue-SF (Petrides, 2009), which captures individuals’ self-perceptions of their emotional functioning. This approach aligns more closely with the study’s focus on learners’ subjective affective experiences within classroom settings.

Trait emotional intelligence typically encompasses several interrelated dimensions, including emotional perception, regulation, utilization, and social competence (Dastgoshadeh & Javanmardi, 2021; Ketabdar et al., 2014; Zhang & Zhang, 2023). These competencies collectively reflect learners’ ability to recognize emotional cues, regulate their internal states, use emotions adaptively in decision-making, and maintain positive interpersonal relationships. In educational contexts, EI has been shown to enhance academic achievement, learning motivation, and self-efficacy, largely by supporting adaptive coping and emotional regulation (Aziz et al., 2020). Beyond education, EI contributes to effective social functioning, leadership, and mental health, but the present study focuses specifically on its implications for second language acquisition, where emotional dynamics play a central role.

However, existing studies suggest that the relationship between EI and L2 WTC is not merely linear, and the underlying mechanisms remain unclear. There are inconsistencies across findings—for instance, L. Fan et al. (2024) found that foreign language enjoyment partially mediates the relationship between EI and L2 WTC, while foreign language anxiety does not; conversely, L. Wei et al. (2022) argued that in classroom social environments, anxiety serves as a critical mediator. According to ecological systems theory (Benner et al., 2008), human behavior results from the dynamic interplay between contextual and individual factors. Thus, the internal mechanisms underlying the relationship between emotional intelligence and second language willingness to communicate warrant further exploration.

In the field of SLA, the significance of emotional intelligence has been widely acknowledged, particularly for its impact on L2 WTC. Studies among EFL learners in countries such as Turkey and Spain have demonstrated a significant positive correlation between EI and L2 WTC, identifying EI as a key antecedent to enhanced communicative willingness (Fernández-García & Fonseca-Mora, 2022; Oz, 2015). Research conducted among university-level EFL learners in China has further supported these findings, revealing that EI can positively influence L2 WTC (L. Fan et al., 2024; L. Wei et al., 2022). However, most existing evidence has been derived from research in public or comprehensive universities. Students in Chinese private colleges, who often experience higher academic pressure and fewer language-learning resources, may exhibit different affective and communicative patterns (X. Chen, 2019; Y. Li & He, 2021). Understanding how emotional intelligence operates within this context can thus provide a more nuanced picture of the emotional dynamics underlying L2 WTC.

From a theoretical perspective, emotional intelligence theory (Salovey & Mayer, 1990) and the Control–Value Theory of achievement emotions (Pekrun, 2006) jointly illuminate why EI should influence communicative willingness. Specifically, EI-related competencies (e.g., accurate emotion perception and effective regulation) enable learners to appraise and manage achievement-related emotions, fostering positive affective states such as enjoyment while attenuating debilitating emotions such as anxiety. According to Control–Value Theory, these achievement emotions, shaped by individuals’ control and value appraisals, directly affect motivation and approach–avoidance tendencies, which in turn influence communicative behaviors (e.g., willingness to initiate or sustain L2 interaction). Thus, EI can be expected to exert both direct effects on WTC and indirect effects mediated by proximal emotions such as foreign language enjoyment.

At the same time, ecological systems perspectives highlight that emotional and communicative processes are embedded within specific sociocultural and institutional contexts (Benner et al., 2008). Within Chinese private colleges, differences in affective climates, instructional practices, and communicative norms may shape how emotional intelligence translates into positive emotions and language use. Such contextual affordances could either strengthen or constrain the pathway from emotional intelligence to foreign language enjoyment and, in turn, to willingness to communicate. Investigating this mechanism in the private college context thus provides valuable evidence on the robustness and contextual sensitivity of emotional mechanisms in L2 communication.

## 3. The Mediating Role of Foreign Language Enjoyment

Foreign Language Enjoyment (FLE) refers to the positive emotional experience that learners derive from engaging in foreign language learning (Dewaele & MacIntyre, 2014). It encompasses three interrelated dimensions: enjoyment from the learning process itself, enjoyment supported by teachers, and enjoyment facilitated by peers (Dewaele et al., 2018). As a core academic emotion, FLE broadens learners’ cognitive and behavioral resources, is associated with greater motivation and engagement, and contributes to well-being and achievement (Elahi Shirvan et al., 2020). The Control-Value Theory of achievement emotions (Pekrun, 2006; Shao et al., 2020) provides a theoretical basis for understanding the role of FLE in shaping L2 WTC. When learners perceive language learning as both controllable and valuable, they are more likely to experience enjoyment, which in turn fosters communicative confidence and readiness (Yin et al., 2024). Empirical evidence consistently shows that FLE positively predicts WTC, whereas anxiety exerts a negative influence (Dewaele et al., 2018; C. Li & Chen, 2018; Y. Q. Wang, 2023). Dewaele (2019) further demonstrated that FLE enhances WTC and academic performance among English learners, partly by mitigating anxiety. Similarly, Botes et al. (2021) emphasized FLE’s central role in promoting communication and emotional resilience in language learning contexts.

A growing body of research has also linked EI with FLE, suggesting that emotionally intelligent learners are better equipped to regulate their affective experiences and sustain enjoyment during learning. For example, Resnik and Dewaele (2020) found that German students with higher EI reported greater classroom enjoyment and lower anxiety. In a Chinese university sample, Z. Chen et al. (2024) similarly showed that trait EI enhances FLE while reducing classroom anxiety. This connection between learners’ EI and their emotional experiences may be particularly pronounced in private Chinese colleges, where contextual pressures, such as limited institutional support and heightened competition, intensify emotional responses during language learning (Q. Wang et al., 2020). Compared with their peers in public universities, students in private institutions often rely more on internal emotional resources to sustain motivation and engagement. Consequently, EI may play a particularly salient role in fostering FLE and, in turn, WTC.

Building on both the Control–Value Theory (Pekrun, 2006) and Ecological Systems Theory (Benner et al., 2008), it is theoretically plausible that unique configurations of EI and FLE constitute differentiated emotional profiles, which may explain variations in students’ willingness to communicate. Despite the growing empirical evidence, measurement practices across studies vary considerably, and some inconsistency in construct operationalization (e.g., abbreviated FLE scales, self-report EI measures) may partly account for mixed findings in the literature.

Recent studies have begun to apply person-centered approaches in language learning contexts. For instance, B. B. Li et al. (2023) employed LPA to identify distinct learner profiles based on thinking patterns and foreign language emotions, revealing that students with a growth mindset typically exhibited higher foreign language enjoyment and lower anxiety. Similarly, Xue and Wang (2022) identified three distinct emotion regulation profiles: over-regulation, moderate regulation with problem confrontation and low regulation with avoidance, each associated with distinct affective experiences.

Given that students in Chinese private colleges often display diverse affective and motivational patterns due to institutional and contextual factors (B. B. Li et al., 2023; Xue & Wang, 2022), integrating both variable-centered and person-centered perspectives allows for a more nuanced understanding of how EI relates to L2 WTC within this specific educational context. Grounded in both ecological and control–value theories, this study employs variable-centered and person-centered analyses to address the following research questions:

1. What is the mediating role of FLE in the relationship between EI and L2 WTC among students from Chinese private colleges?
2. What are the distinct typologies of learners based on different combinations of EI and FLE?
3. How and why do theoretically derived EI–FLE learner profiles differ in their L2 WTC?

## 4. Method

### 4.1. Participants

Following the recommendation of Kretzschmar and Gignac (2019), a minimum sample size of 490 participants was deemed necessary to achieve adequate statistical power (≥0.80) for detecting small-to-medium effect sizes (*f*^2^ ≈ 0.05) in mediation and latent profile models with multiple observed indicators. Ultimately, 1387 questionnaires were distributed through convenience sampling, targeting non-English-major students from two private colleges in Hubei Province, China. These institutions were selected based on their accessibility and representativeness of typical private college English programs in the region. Within each college, students were invited to participate voluntarily during class sessions. Only full-time students who provided complete and consistent responses were retained for analysis. Of the 1387 questionnaires distributed, 1111 were valid, yielding a response rate of 80.10% (minor numerical difference due to rounding). To examine potential non-response bias, early and late respondents were compared across key demographic variables (e.g., age, gender, and academic year). No significant differences were found (*p* > 0.05), suggesting minimal non-response bias and acceptable representativeness of the sample. Participants represented a range of academic years: 633 first-year students (57.0%), 388 second-year students (34.9%), 52 third-year students (4.7%), and 38 fourth-year students (3.4%). The predominance of first-year students aligns with the instructional structure of Chinese private colleges, where English courses are mainly required during the first two years, while upper-year students focus on discipline-specific coursework and internships. Consequently, data collection conducted through English classes naturally involved a larger proportion of first-year participants. The mean age of the participants was 19.03 years (*SD* = 1.43), and the gender distribution was relatively balanced, with 532 males (47.9%) and 579 females (52.1%).

### 4.2. Measures

All instruments were administered in Chinese to minimize potential comprehension bias, and validated Chinese versions were used where available.

#### 4.2.1. Emotional Intelligence

EI was assessed using the simplified Chinese version of the Trait Emotional Intelligence Questionnaire–Short Form (TEIQue-SF; Petrides, 2009), which was adapted by C. C. Li (2020) for Chinese university students. This adaptation aimed to enhance linguistic clarity and cultural appropriateness while preserving the original four-dimensional structure (emotionality, self-control, sociability, and well-being). The Chinese version has demonstrated satisfactory reliability and construct validity in previous validation studies with Chinese college samples (Feher et al., 2019). Participants rated 30 items on a 7-point Likert scale. Confirmatory factor analysis (CFA) indicated a good model fit: *χ*^2^(250, *N* = 1111) = 504.03, *χ*^2^*/df* = 2.016, *RMSEA* = 0.030, *CFI* = 0.955, *GFI* = 0.952, and *TLI* = 0.983. In the present study, the scale demonstrated excellent internal consistency, with a Cronbach’s alpha of 0.971.

#### 4.2.2. Willingness to Communicate in a Second Language

L2 WTC was assessed using the 10-item scale developed by Peng and Woodrow (2010). Each item was rated on a 5-point Likert scale ranging from 1 (“strongly disagree”) to 5 (“strongly agree”). CFA results supported a good model fit: *χ*^2^*/df* = 2.027, *RMSEA* = 0.029, *CFI* = 0.956, *GFI* = 0.949, and *TLI* = 0.981. The Cronbach’s alpha for this scale in the current study was 0.903, indicating high reliability.

#### 4.2.3. Foreign Language Enjoyment

FLE was measured using the Foreign Language Enjoyment Scale developed by Jin and Zhang (2019). The scale comprises 16 items across three subdimensions: enjoyment of teacher support, enjoyment of learning, and enjoyment of peer support. All items were retained in the present study to assess students’ overall enjoyment of learning English. The instrument was reviewed by bilingual language education experts and pilot tested to ensure linguistic clarity and cultural relevance for Chinese EFL learners. Participants responded on a 5-point Likert scale ranging from 1 (“strongly disagree”) to 5 (“strongly agree”). Confirmatory factor analysis (CFA) supported the three-factor structure, indicating an excellent model fit to the data: *χ*^2^(101, N = 1111) = 231.47, *χ*^2^*/df* = 2.29, *RMSEA* = 0.034, *CFI* = 0.982, *GFI* = 0.958, and *TLI* = 0.977. The overall scale demonstrated high internal consistency (Cronbach’s *α* = 0.925), with satisfactory reliability for each subdimension: teacher support (*α* = 0.901), learning (*α* = 0.908), and peer support (*α* = 0.884).

### 4.3. Data Analysis

Preliminary analyses examined the effects of gender, age, and academic year. As none showed significant associations with the main study variables (*ps* > 0.05), they were not included as covariates in subsequent analyses. All statistical analyses were performed using SPSS 27.0 and Mplus 8.3. Descriptive statistics, correlation analyses, and mediation testing were conducted in SPSS, with the mediation model estimated using PROCESS macro (Model 4). LPA and model fit evaluations were conducted in Mplus. Prior to analyses, the dataset was screened for data entry errors, missing values, and outliers. Cases with more than 2% missing responses were excluded through listwise deletion, and the remaining minimal missing data were treated with pairwise deletion. The assumptions of normality, linearity, and homoscedasticity were examined through skewness, kurtosis, and residual plots, with no substantial violations detected. The dataset met key assumptions for multivariate analyses. Multicollinearity diagnostics indicated acceptable tolerance values (>0.10) and VIFs (<3.0), suggesting no serious multicollinearity among predictors.

Mediation analyses were performed with the PROCESS macro (version 4.1). Indirect effects were estimated using 5000 bootstrap samples and 95% bias-corrected confidence intervals to ensure robust inference.

Latent profile analysis was conducted in Mplus 8.3 using maximum likelihood estimation with robust standard errors (MLR). Each model was estimated with 500 random sets of starting values and 100 final-stage optimizations, and convergence was confirmed when the best log-likelihood value was replicated. Model adequacy was evaluated using AIC, BIC, aBIC, entropy, and the Lo–Mendell–Rubin likelihood ratio test.

### 4.4. Procedure

Data were collected in July 2024 during regular English classes. Participants completed the paper-based questionnaires in approximately 20 min under the supervision of trained research assistants. Participation was voluntary and anonymous, and informed consent was obtained prior to data collection. The study protocol was reviewed and approved by the institutional Ethics Committee in accordance with the relevant guidelines and regulations.

## 5. Results

### 5.1. Test for Common Method Bias

To minimize potential common method bias (CMB), several procedural remedies were implemented during data collection, such as ensuring participant anonymity, randomizing item order, and embedding lie-detection items. In addition, Harman’s single-factor test was conducted, and the first unrotated factor accounted for 32.97% of the total variance, below the commonly accepted threshold of 40% (Zhou & Long, 2004).

Considering that Harman’s test alone has been criticized as insufficient to rule out CMB (Podsakoff et al., 2003, 2012), a confirmatory single-factor model was further tested. The CFA results showed a poor model fit (*χ*^2^*/df* = 8.21, *RMSEA* = 0.105, *CFI* = 0.658, *TLI* = 0.624), indicating that common method bias was not a serious concern in this study.

### 5.2. Descriptive Statistics and Correlation Analysis

Means, standard deviations, and intercorrelations among all study variables are presented in Table 1. Correlational analyses revealed significant positive relationships among EI and FLE (*r* = 0.24, *p* < 0.001), EI and L2 WTC (*r* = 0.35, *p* < 0.001), and FLE and L2 WTC (*r* = 0.63, *p* < 0.001).

Although the correlation between FLE and L2 WTC was relatively high, this association aligns with theoretical expectations, as both reflect learners’ positive affect and engagement in the language classroom. Confirmatory factor analysis supported the discriminant validity of the three constructs (*χ*^2^*/df* = 2.45, *RMSEA* = 0.045, *CFI* = 0.957, *TLI* = 0.948). The three-factor model fitted the data substantially better than a one-factor model, indicating that the variables represent distinct constructs. The moderate correlations between EI and the other variables likely reflect differences in scale length and construct breadth.

### 5.3. Variable-Centered Analysis of EI, FLE and L2 WTC

**Figure 1:**
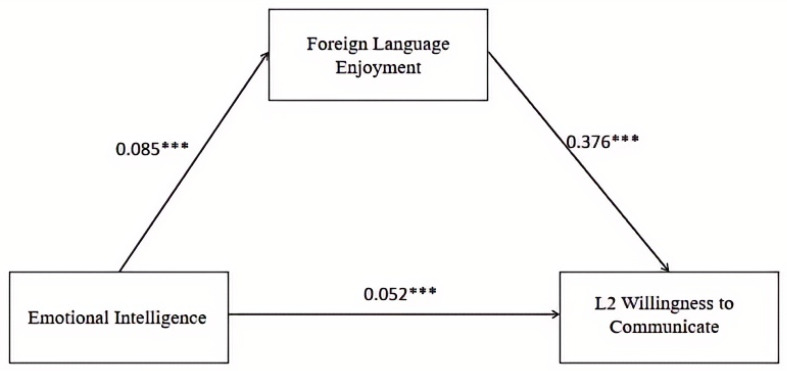
Mediating Role of Foreign Language Enjoyment Between Emotional Intelligence and L2 Willingness to Communicate. Note. *** *p* < 0.001.

To examine the relationships among EI, FLE, and L2 WTC, we conducted a mediation analysis using PROCESS macro (Model 4), with WI as the predictor, FLE as the mediator, and L2 WTC as the outcome variable. As shown in Figure 1, EI significantly predicted FLE (*β* = 0.085, *p* < 0.001), and foreign language enjoyment significantly predicted L2 WTC (*β* = 0.376, *p* < 0.001).

To further examine the mediating effect, the bias-corrected percentile bootstrap method with 5000 resamples was employed to calculate the 95% confidence intervals. Note that the coefficients reported in Table 2 are standardized regression estimates, which are not directly comparable to the zero-order correlations shown in Table 1. As shown in Table 2, the indirect effect of emotional intelligence on L2 willingness to communicate through foreign language enjoyment was significant (b = 0.032, 95% CI [0.024, 0.040]), accounting for 38.30% of the total effect. Both unstandardized and completely standardized coefficients are reported, along with the corresponding Cohen’s d values, to provide a clearer interpretation of the magnitude of the mediation effects. These results indicate that FLE partially mediates the relationship between EI and L2 WTC among non-English major students from Chinese private colleges, thereby addressing Research Question 1.

### 5.4. Person-Centered Analysis of EI, FLE and L2 WTC

To further explore Research Question 2, latent profile analysis (LPA) was conducted. Models with one to five classes were estimated and compared using multiple fit indices, including the Akaike Information Criterion (AIC), Bayesian Information Criterion (BIC), adjusted BIC (aBIC), and entropy. Smaller AIC, BIC, and aBIC values indicate better model fit, and an entropy value of 0.80 or higher suggests adequate classification accuracy. In addition, the Lo–Mendell–Rubin test (LMRT) and the Bootstrap Likelihood Ratio Test (BLRT) were used to determine whether a model with k classes fit significantly better than a model with k–1 classes (Morgan et al., 2016).

As shown in Table 3, although the AIC, BIC, and aBIC values continued to decrease for the 4- and 5-class models, the reductions were marginal (ΔBIC ≈ 317 between the 3- and 4-class models, and ≈213 between the 4- and 5-class models), suggesting limited improvement in model fit. The 3-class model exhibited high entropy (0.960) and yielded interpretable, theoretically meaningful profiles. In contrast, the 4- and 5-class models produced small, redundant subgroups (<6% of the sample) with minimal conceptual distinction. Therefore, the 3-class solution was selected as optimal, balancing statistical adequacy, parsimony, and interpretability.

**Figure 2:**
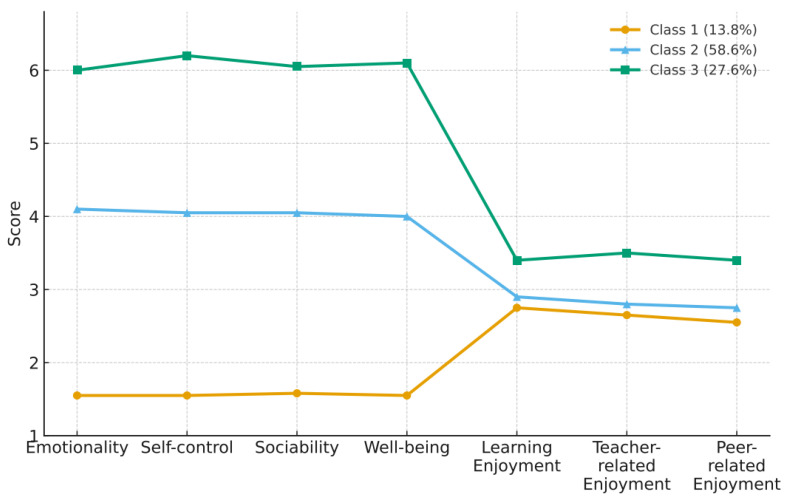
Latent Profile Plot of Emotional Intelligence and Foreign Language Enjoyment.

As shown in Figure 2, Class 1 (13.8% of the sample) displayed the lowest scores across all four dimensions of EI and three dimensions of foreign language enjoyment and was therefore labeled the “Low EI–Low Enjoyment” group. Class 2 (58.6%) scored at moderate levels on all dimensions and was named the “Moderate EI–Moderate Enjoyment” group. Class 3 (27.6%) showed the highest levels of both emotional intelligence and foreign language enjoyment and was thus termed the “High EI–High Enjoyment” group.

As shown in Table 4, a one-way ANOVA was conducted to examine group differences in L2 willingness to communicate across the three latent profiles. The results indicated a statistically significant overall difference, *F*(2, 1108) = 6.091, *p* < 0.005, *η*^2^ = 0.002. However, following Cohen (1988) guidelines, this effect size is considered trivially small, suggesting that the practical magnitude of these differences was minimal despite statistical significance due to the large sample size (N = 1111). Post hoc comparisons revealed a consistent trend (C3 > C2 > C1), indicating that learners in the high EI–high enjoyment profile reported slightly higher willingness to communicate than those in the other groups.

## 6. Discussion

This study integrated both variable-centered and person-centered approaches to comprehensively examine the relationships among emotional intelligence, foreign language enjoyment, and willingness to communicate in a second language among students from Chinese private colleges. From the variable-centered perspective, the findings revealed that EI significantly predicted L2 WTC, with FLE playing a partial mediating role. These results underscore the pivotal role of EI in second language learning and highlight the bridging function of FLE.

From the person-centered perspective, LPA identified three distinct subgroups based on students’ EI and FLE profiles. These profiles reflected significant individual differences and enriched the understanding of how variations in EI and FLE are associated with differing levels of L2 WTC among students. Together, these two perspectives complement each other, illustrating both the average-level mechanism and the individual-level differentiation in this emotional–communicative relationship.

### 6.1. Variable-Centered Perspective

The variable-centered analysis indicated that EI positively predicts college students’ L2 WTC, aligning with the findings of Birjandi and Hadidi Tamjid (2012) and Pishghadam and Ghonsooli (2008), and highlighting the critical value of emotional intelligence in L2 learning. From an ecological perspective on L2 classrooms (Benner et al., 2008), language acquisition and development occur through social engagement, and emotional intelligence, an essential psychological trait, influences individuals’ ability to perceive and regulate emotions. Students with high emotional intelligence are more adept at detecting emotional cues and communicative intentions during L2 interactions, allowing them to adjust their verbal and nonverbal behaviors flexibly to enhance communication.

For example, during a social gathering with international peers, such students may accurately interpret conversation topics and emotional states through facial expressions and body language, enabling smoother interactions. These successful experiences can increase their willingness to participate in similar activities in the future, bringing a sense of accomplishment and satisfaction (K. Fan & Wang, 2023). This finding supports the perspective of MacIntyre et al. (1998), which emphasizes the motivational role of emotional intelligence in the formation of L2 willingness to communicate. Thus, enhancing students’ emotional intelligence may serve as an effective strategy for promoting L2 communication. Both school and family education should prioritize emotional intelligence development through targeted curricula and activities, helping students navigate complex social interactions and fostering communicative confidence and enjoyment. Moreover, foreign language enjoyment was found to mediate the relationship between emotional intelligence and L2 willingness to communicate. This result is consistent with numerous prior studies (e.g., Dewaele & MacIntyre, 2014; C. C. Li, 2020; Kruk et al., 2023) and further corroborates the propositions of L. Fan et al. (2024). Students with higher levels of foreign language enjoyment tend to exhibit stronger willingness to engage in L2 communication. Immersed in a positive emotional atmosphere, they are intrinsically motivated to participate in various L2 communicative contexts. This engagement not only facilitates the development of their communicative skills but also improves their oral fluency.

According to the control–value theory of achievement emotions (Pekrun, 2006), positive academic emotions such as enjoyment arise when learners appraise a learning activity as both valuable and controllable. In the context of L2 communication, emotionally intelligent students (Salovey & Mayer, 1990) are better able to regulate their affective responses and sustain feelings of enjoyment. This enjoyment enhances cognitive engagement, promotes flexible and creative language use, and encourages learners to take communicative risks. From an ecological perspective (Benner et al., 2008), such positive emotional experiences also contribute to a supportive classroom atmosphere that nurtures learners’ willingness to communicate, establishing a dynamic and mutually reinforcing cycle between emotion and communication.

Overall, these findings reveal that emotional intelligence influences L2 willingness to communicate both directly and indirectly through foreign language enjoyment. This mediation pattern captures the average-level tendencies among learners, illustrating how emotional and motivational factors operate in tandem to shape communicative behavior. In line with the study’s dual analytical approach, this mechanism also extends to the person-centered level, where individual differences in emotional profiles may lead to variations in the strength of this mediation process.

### 6.2. Person-Centered Perspective

This study employed latent profile analysis (LPA) to explore potential subgroups of college students based on their combinations of emotional intelligence and foreign language enjoyment, and further examined differences in L2 willingness to communicate across these profiles. Based on a comprehensive assessment of model fit indices and interpretability, three distinct profiles were identified: High EI–High Enjoyment, Moderate EI–Moderate Enjoyment, and Low EI–Low Enjoyment.

The results revealed a clear covariation pattern between emotional intelligence and foreign language enjoyment, suggesting a consistent alignment in their levels (i.e., low–low, moderate–moderate, high–high). Students in the Low EI–Low Enjoyment group demonstrated significantly lower levels of willingness to communicate in L2 compared to the other two groups. Although this subgroup represented a relatively small proportion of the overall sample (13.8%), their low L2 WTC is a point of concern that warrants attention. In contrast, the High EI–High Enjoyment group, comprising 27.6% of the participants, showed the highest levels of L2 willingness to communicate. The Moderate EI–Moderate Enjoyment group made up the largest proportion (58.6%) and exhibited moderate levels of L2 WTC.

These results offer empirical support for the revised RQ3—namely, that theoretically meaningful EI–FLE learner profiles differ systematically in their willingness to communicate. Drawing on the control–value theory (Pekrun, 2006) and ecological systems theory (Benner et al., 2008), such differences can be understood as the result of variations in learners’ emotional regulation capacities and perceived control–value appraisals, which together shape emotional experiences and communicative behaviors.

These findings suggest the importance of targeted interventions for different student profiles. Specifically, students in the Low EI–Low Enjoyment group should be prioritized for emotional intelligence training and strategies to enhance foreign language enjoyment, with the aim of fostering greater willingness to communicate. Meanwhile, for students in the Moderate EI–Moderate Enjoyment group, personalized programs aimed at further improving their emotional and affective resources could help facilitate their transition into the High EI–High Enjoyment group.

Overall, these person-centered findings reinforce the conclusions drawn from the variable-centered analysis—namely, that individual differences in emotional intelligence positively predict foreign language enjoyment, which in turn positively influences L2 willingness to communicate. This aligns with previous research (e.g., Song & Wang, 2024) and further underscores the synergistic role of emotional intelligence and enjoyment in promoting communicative engagement in second language in the context of Chinese private colleges.

### 6.3. Integration of Variable- and Person-Centered Findings

Taken together, the findings from the variable-centered and person-centered analyses provide complementary insights into the complex interplay among emotional intelligence, foreign language enjoyment, and L2 willingness to communicate. The mediation model clarified the average-level mechanism, showing that emotionally intelligent learners tend to experience greater enjoyment, which in turn fosters higher willingness to communicate. Meanwhile, the latent profile analysis revealed that this mechanism may not operate uniformly across all learners. Rather, distinct emotional–motivational configurations (e.g., High EI–High Enjoyment vs. Low EI–Low Enjoyment) highlight meaningful subgroup variations in how emotional intelligence and enjoyment jointly shape communicative behavior. Integrating these perspectives, the current study suggests that while the mediating effect of enjoyment reflects a general tendency at the population level, the strength and manifestation of this mechanism differ across learner profiles. This integration underscores the importance of considering both between-person variability and within-person processes when examining affective and communicative outcomes in second language learning. In the context of Chinese private colleges, these findings also shed light on how emotional and motivational dynamics operate among non-English-major students who often face limited language exposure and communicative opportunities outside the classroom.

Despite these theoretical insights, the practical implications appear more nuanced. Although higher emotional intelligence and enjoyment are associated with greater willingness to communicate, their overall influence is limited in magnitude. Emotional factors may function as facilitators or background conditions that support communication rather than as decisive determinants. Therefore, pedagogical efforts to enhance L2 willingness to communicate should not rely solely on fostering positive emotions; instead, they should integrate affective support with task design, classroom interaction, and contextual engagement.

Nevertheless, the findings should be interpreted with caution given the self-report nature of the measures and the correlational design, which preclude firm causal inferences.

### 6.4. Future Directions

To deepen understanding of the mechanisms underlying the identified learner profiles, future research could adopt an exploratory mixed-methods design that incorporates qualitative approaches such as interviews, classroom observations, or reflective journals. Such methods would allow researchers to examine why and how learners with different emotional intelligence–enjoyment configurations develop distinct patterns of willingness to communicate. Integrating qualitative insights with quantitative findings could yield a more holistic account of the dynamic emotional and communicative processes shaping second language learning.

## 7. Conclusions

Grounded in emotional intelligence theory, ecological systems theory and the control–value theory of achievement emotions, this study integrated both variable-centered and person-centered approaches to investigate the relationships among emotional intelligence, foreign language enjoyment, and willingness to communicate in a second language among students from Chinese private colleges. The findings revealed that emotional intelligence significantly and positively predicted L2 WTC, with FLE serving as a partial mediator in this relationship at both the variable-centered and person-centered levels.

Through latent profile analysis, three distinct emotional intelligence–enjoyment profiles were identified: a High EI–High Enjoyment group, a Moderate EI–Moderate Enjoyment group, and a Low EI–Low Enjoyment group. Significant differences in L2 WTC were observed across these subgroups, with students in the High EI–High Enjoyment profile exhibiting the strongest willingness to communicate. These findings provide theoretically grounded evidence that learners’ emotional configurations, rooted in their capacity for emotional regulation and enjoyment, shape their communicative engagement in L2 contexts.

From a pedagogical perspective, the results underscore the importance of cultivating learners’ emotional intelligence and fostering positive classroom climates to enhance students’ communicative confidence and engagement. Teachers in Chinese private colleges are encouraged to design affectively supportive learning environments that integrate emotion regulation training, enjoyable classroom activities, and interactive communication tasks.

Several limitations should be acknowledged. First, the cross-sectional design restricts the ability to capture dynamic developmental changes over time. Moreover, the predominance of first-year participants may reflect the curricular structure of Chinese private colleges, where compulsory English instruction is typically concentrated in the first two academic years. As emotional intelligence and communicative confidence are likely to evolve with academic experience, future research should consider employing longitudinal designs, such as latent transition analysis or cross-lagged panel models, to examine developmental trajectories and causal relationships across academic stages.

## Figures and Tables

**Table 1:** Descriptive Statistics and Correlations Among Variables (N = 1111).

	*M*	*SD*	1	2	3
Emotional Intelligence	130.59	44.04	1		
Foreign Language Enjoyment	48.57	15.99	0.24 ***	1	
L2 Willingness to Communicate	30.33	10.48	0.35 ***	0.66 ***	1

Note. *** *p* < 0.001 (two-tailed). All variables are presented as summed scores rather than mean item ratings.

**Table 2:** Mediation Analysis Results.

Path	Indirect Effect	BootSE	BootLLCI	BootULCI	Completely Standardized Effect	Cohen’s *d*	Proportion Mediated (%)
Total Effect	0.084	0.006	0.071	0.097	0.352	0.75	—
Direct Effect	0.052	0.005	0.041	0.063	0.217	0.45	61.70%
Indirect Effect	0.032	0.004	0.024	0.040	0.135	0.27	38.30%

Note. BootSE = bootstrapped standard error. BootLLCI = bootstrapped lower limit of confidence interval; BootULCI = bootstrapped upper limit of confidence interval. Completely standardized effects and corresponding Cohen’s *d* are provided for interpretability. 95% confidence intervals that do not include zero indicate significant mediation effects. “—“ indicates not applicable.

**Table 3:** Latent Profile Model Fit Indices for Emotional Intelligence.

Model	AIC	BIC	aBIC	Entropy	LMRT	BLRT	Class Probabilities(%)
1	27,829.435	27,899.617	27,855.149	——	——	——	——
2	26,024.185	26,134.472	26,064.594	0.968	<0.01	<0.01	85.78/14.22
3	24,185.318	24,335.709	24,240.421	0.960	<0.01	<0.01	13.77/58.95/27.28
4	23,818.396	24,008.891	23,888.193	0.885	<0.01	<0.01	13.77/17.55/41.94/26.73
5	23,565.384	23,795.982	23,649.875	0.888	<0.05	<0.01	13.68/14.67/36.36/10.08/25.21

Note. “——” indicates not applicable for the single-class model.

**Table 4:** Differences in L2 WTC Across Latent Profiles (*M* ± *SD*).

Group	L2 WTC		*F*	*η* ^2^	Post Hoc (*p* < 0.005)
Low EI–Low Enjoyment (C1)	4.374	1.343	6.091	0.002	C3 > C2 > C1
Moderate EI–Moderate Enjoyment (C2)	4.117	2.022			
High EI–High Enjoyment (C3)	4.507	1.785			

## Data Availability

All research data are available from the corresponding author upon reasonable request.
